# The health and wellbeing of Australian social housing tenants compared to people living in other types of housing

**DOI:** 10.1186/s12889-023-17267-2

**Published:** 2023-11-24

**Authors:** Megan Freund, Matthew Clapham, Jia Ying Ooi, David Adamson, Allison Boyes, Robert Sanson-Fisher

**Affiliations:** 1 Health Behaviour Research Collaborative, College of Health, Medicine and Wellbeing, University of Newcastle, Callaghan, New South Wales (NSW), 2308 Australia; 2 Equity in Health and Wellbeing Research Program, Hunter Medical Research Institute, New Lambton Heights, NSW, Australia; 3 Clinical Research Design and Statistics Support Unit, Hunter Medical Research Institute, New Lambton Heights, NSW, Australia; 4 Home in Place Co Ltd., Newcastle West, NSW, Australia; 5 University of South Wales, Cardiff, South Wales, UK

**Keywords:** Public housing, Housing and health, Health inequalities, Health risk behaviours, Chronic disease

## Abstract

**Background:**

Although social housing provides access to safe and affordable housing, recent studies have found that social housing tenants consistently have lower levels of health and well-being compared to other people. Given this, there is a need to examine multimorbidity for social housing tenants.

**Methods:**

Secondary data analysis of the 2017-18 Australian National Health Survey (n = 14,327) compared the health of adults residing in social housing compared to people in other housing types (private rentals, homeowners, and homeowners/mortgagees).

**Results:**

Most health factors examined were more prevalent in social housing tenants compared to those living in other housing types. Individual health problems identified as more highly prevalent in social housing tenants compared to all other housing types included mental health issues (43%), arthritis (36%), back problems (32%), hypertension (25%), asthma (22%) and COPD (11%). 24% of social housing tenants reported five or more health factors compared to 3–6% of people in other housing types.

**Conclusions:**

Although these findings are not unexpected, they provide more detailed evidence that social housing providers and policy makers should consider when planning future initiatives.

## Background

Social housing provides access to safe, secure and affordable housing for those who are unable to access suitable accommodation in the private rental market [1]. Social housing is provided by government and increasingly by not-for-profit Housing Associations (UK) and Community Housing Organisations (Australia). In its initial phases, social housing was a response to public health issues identified with the slums accumulated during the industrial revolution, particularly in the United Kingdom. More recently, in Australia and elsewhere, the social housing sector has experienced a long decline characterised by a general underinvestment [2]. Given the scarcity of social housing compared to demand, a process of ‘residualisation’ has occurred. Allocations to social housing are largely provided to highly vulnerable individuals including those with physical and psychosocial disability, those experiencing homelessness or at risk of homelessness, and on very low incomes [1, 3].

Given this pattern of occupancy, it is not surprising that social housing tenants are consistently found to have lower levels of health and well-being compared to other people [4, 5]. However, research investigating the health of social housing tenants often focuses on single elements of health (e.g. obesity, cardiovascular disease or mental ill-health) rather than providing a profile of tenants overall health [6, 7]. Multiple health problems experienced by an individual (i.e., multimorbidity) is associated with lower health related quality of life, higher utilization of health care services and prescribed medications, increased disability, and mortality [8–10]. Further, risky health behaviour (e.g. smoking, alcohol misuse) can increase the likelihood of multimorbidity, and worsen health outcomes [11].

Recent studies of the prevalence of multimorbidity [8, 10, 12, 13], found higher rates were experienced by people from lower socio-economic backgrounds [10, 13]. However, these studies did not examine the health divide experienced by social housing tenants, as distinct from the general low-income population. Addressing the health and wellbeing needs of tenants is an increasingly essential and resource-intensive part of social housing providers’ remit [14]. To enable social housing providers and their partner support agencies to effectively deliver person-centred care, there is a need to examine multimorbidity for social housing tenants specifically. Such information will help inform policy and other decision makers regarding the resources required by social housing providers to effectively address tenant’s heath needs.

The current study aimed to examine, using the 2017-18 Australian National Health survey data, the overall health and wellbeing burden of Australian adults residing in social housing compared to that of people living in other housing types (owner/ owner mortgage/private rental).

## Methods

### Study design

This is a secondary data analysis of the National Health Survey (NHS) 2017-18 dataset [15]. The NHS is an Australia-wide household-based health survey conducted at three-year intervals under the auspices of the Australian Bureau of Statistics (ABS). The NHS data is collected using a stratified multi-level sampling methodology to ensure all sections of the population living in private dwellings within the geographic scope of the survey are represented by the sample (excluding very remote and Indigenous Communities). The survey was implemented by trained ABS interviewers using Computer Assisted Personal Interviews. The survey collected a range of health-related information about Australians aged two years and above. Details regarding the survey are provided in the NHS Users’ Guide for 2017-18 [16].

### Study sample

The 2017-18 NHS included a sample of approximately of 21,315 persons from 16,384 private dwellings across Australia(Australian Bureau of Statistics, 2018). All adults completing the 2017–2018 NHS survey and who provided information on their housing type were eligible to be included in the study. The housing type of participants was determined by information provided to questions about dwelling tenure and/or landlord type. Outright homeowners answered “yes” to the question “Is this dwelling owned or partly owned by you?”. Homeowners/mortgagees answered “yes” to the question “Do you currently have any mortgages or secured loans on this dwelling?”. Private renters answered “yes” to the question “Is this dwelling rented by you?”. Social housing residents nominated their landlord type as a state or territory housing authority.

### Outcome measures

Self-assessed health was measured by one item asking “In general would you say that your health is excellent, very good, good, fair or poor?”

Psychological distress was measured by the 10-item Kessler Psychological Distress Scale (K10) [17]. Respondents indicated how often they have experienced symptoms of anxiety and depression in the past four weeks (all of the time, most of the time, some of the time, a little of the time, none of the time). The K10 is scored from 10 to 50, with higher scores indicating higher levels of distress. Scores can be categorised as: low levels of distress (score 10–15); moderate levels of distress [15–20]; high levels of distress [21–28]; and very high levels of distress (30–50) [17].

Disability was based on whether the participant had a current condition that has lasted or was expected to last for six months or more, and if the condition had impacted their ability regarding movement and transport or their engagement in employment and education. The conditions assessed were shortness of breath, chronic or recurring pain, a nervous or emotional condition, or long-term effects as a result of a head injury, stroke or other brain damage. Participants were also asked about any other conditions they have that has lasted six months or more. Each participant was allocated to one of six categories of disability: profound (always need help with self-care, mobility and communication), severe (do not always but may require help at times), moderate (have difficulty with the self-care, mobility and communication), mild (simply required aids to undertake self-care, mobility and communication or are unable to do any of the additional mobility tasks [e.g. easily walk 200 m]), school/employment restriction only (having a difficulty with school/study or work), or no disability.

Selected medical long-term conditions. Participants were asked if they have ever been told by a doctor or nurse that had any of the following long-term conditions (yes/no/don’t know): asthma, cancer, cardiovascular disease, arthritis, osteoporosis, diabetes, kidney diseases or mental, behavioural or cognitive conditions.

Other long-term medical conditions. Participants were also asked if they had any other conditions (not assessed as part of the selected medical conditions) that lasted or were expected to last for six months or more. The five most prevalent conditions across all housing types were listed.

Overweight/obese. Participant height (centimetres) and weight (kilograms) were measured and each participant’s body mass index (BMI) was calculated with a BMI score of ≥ 25 kg/m^2^ classified as overweight or obese [18].

Health risk behaviours assessed were smoking, alcohol misuse, inadequate fruit and vegetable consumption, and inadequate physical activity. Current daily smokers were identified by asking “On average, on how many days do you smoke per week?”. Participants at risk of long-term harm (seven-day average drinking exceeded 2009 guidelines) [19] or short-term harm (consumed 5 or more alcoholic drinks on any one occasion) from alcohol misuse were identified. Survey participants were asked to report the number of serves of fruit and serves of vegetables they usually consumed each day and classified as having met or not met the 2013 NHMRC guidelines for fruit and vegetable consumptions separately [20]. Physical activity was assessed by three survey items related to the number of times, minutes and intensity of physical activities over the last week. Participants met the 2014 Physical Activity Guidelines, Australia’s Physical Activity and Sedentary Behaviour Guidelines for the participant’s age group [21].

Demographic characteristics included age, gender, employment status, education level, whether mainly spoke English at home and household composition (i.e., who the participant lives with).

### Analysis

The demographic characteristics and the prevalence for each health burden factor by housing type were reported using descriptive statistics. Postcode was used to calculate the Socio-Economic Indexes for Areas (SEIFA) [22] and Accessibility and Remoteness Index of Australia (ARIA+) [23].

#### Association of heath burden factor with housing type

Multiple logistic regression was used to assess the association between housing type and each health burden factor. To enable this, each health burden factor responses were dichotomised based on the authors’ perspective of impact on overall health burden and/or the distribution of the data. The variables were dichotomised as follows: self-assessed health (fair/poor versus good/very good/excellent); level of psychological distress (high/very high versus low/moderate levels; level of disability (profound/severe/moderate disability versus mild disability/no limitations); number of selected long-term conditions (three or more conditions versus two or fewer); other long-term conditions (any other long-term versus none); weight (overweight/obese versus underweight/normal weight); and number of health risk behaviours (three or more vs. two or less).

#### Association of overall heath burden score with housing type

An overall burden score was calculated for each housing type. The endorsement of the following contributed one point to the burden score: having fair or poor self-assessed health; high or very high distress levels; profound, severe or moderate disability; reporting three or more selected health conditions; reporting any other health condition; being overweight or obese; and reporting three or more health risk behaviours. Ordinal logistic regression was used to assess the association between housing type and the overall burden score. The score ranged from zero (lowest health burden) to seven (highest health burden). Scores six and seven were combined to assist with model fit.

All health burden prevalence and analyses are reported as population weighted values, and standard errors and p-values were calculated with 60 replicate weights using the Jacknife variance method to account for survey design. All regression models were controlled for age, gender, SEIFA (IRSD) 2016 and ARIA + 2016. Age and SEIFA were modelled using natural cubic splines with knots at percentiles 5, 27.5, 50, 72.5 and 95%. Odds ratios (OR), 95% confidence intervals and p-values are provided. Analysis was conducted using the ABS remote access DataLab. All analyses were undertaken using SAS software, Version 9.4 (SAS Institute, Cary, North Carolina, USA) [24].

## Results

### Sample description

Data for 16,370 adults were included in National Health Survey data and 14,327 of these participants provided information on their housing type (i.e., constituted the study sample). Of these, 678 (4.7%) were in social housing, 2,764 (19.3%) were in private rentals, 5,429 (38.0%) were homeowners, and 5,456 (38.1%) were homeowners/mortgagees. The population weighted characteristics of the survey sample by housing type is provided in Table 1.

**Table 1:** Sample sociodemographic characteristics (N = 16,381,935 weighted)

Characteristic	Social housing (N = 441,963, 2.7%) % (SE)^a^	Private rental (N = 3,316,040, 20.2%) % (SE)^a^	Homeowner (N = 5,738,797, 35.0%) % (SE)^a^	Homeowner/ mortgagee (N = 6,885,135, 42.0%) % (SE)^a^
**Age**				
18–24	12% (2.54)	17% (0.99)	8% (0.59)	11% (0.50)
25–34	11% (1.86)	35% (1.16)	6% (0.50)	20% (0.55)
35–44	9% (1.54)	23% (0.86)	6% (0.39)	25% (0.44)
45–54	16% (1.85)	14% (0.64)	12% (0.47)	23% (0.42)
55–64	22% (2.07)	6% (0.43)	22% (0.45)	15% (0.46)
65–74	16% (1.94)	3.4% (0.33)	27% (0.66)	4.1% (0.24)
75+	12% (1.43)	1.1% (0.19)	20% (0.58)	1.0% (0.20)
**Gender**				
Female	54% (3.00)	51% (1.11)	53% (0.66)	51% (0.65)
**Employment**				
Employed	19% (2.12)	75% (0.98)	42% (0.80)	84% (0.72)
Unemployed	10% (1.54)	4.0% (0.51)	1.9% (0.28)	2.4% (0.31)
Not in labour force	70% (2.54)	22% (0.92)	56% (0.77)	13% (0.59)
**Education**				
University	7% (1.52)	32% (1.12)	24% (0.95)	35% (0.75)
Diploma/Certificate	23% (2.18)	32% (1.13)	29% (0.77)	34% (0.70)
Year 12	15% (2.51)	17% (0.91)	12% (0.67)	15% (0.67)
Year 10 or 11	24% (2.17)	11% (0.77)	17% (0.56)	10% (0.50)
Lower/Not determined	31% (2.50)	7% (0.56)	17% (0.54)	6% (0.44)
**SEIFA**				
1–2 (lowest)	34% (3.62)	20% (1.07)	16% (0.79)	14% (0.83)
3–4	20% (2.64)	20% (1.32)	21% (1.09)	19% (1.09)
5–6	26% (4.02)	20% (1.36)	20% (0.81)	22% (1.12)
7–8	14% (1.99)	21% (1.33)	19% (0.87)	23% (0.96)
9–10 (highest)	6% (1.36)	19% (1.13)	23% (0.99)	22% (0.93)
**ARIA+**				
Major cities	70% (3.31)	79% (0.87)	69% (0.84)	74% (0.53)
Inner regional	13% (2.18)	15% (0.82)	21% (0.72)	18% (0.52)
Outer regional	14% (2.42)	6% (0.55)	9% (0.48)	7% (0.32)
Remote	2.7% (0.78)	0.4% (0.09)	1.4% (0.31)	1% (0.18)
**Mainly speaks English at home**				
Yes	83% (2.97)	78% (1.07)	90% (0.66)	87% (0.63)
**Housing composition**				
Lives alone	43% (3.00)	15% (0.69)	19% (0.56)	8% (0.34)
Lives with adults & children	16% (2.44)	35% (1.36)	17% (0.68)	54% (0.96)
Lives with children only	13% (1.57)	6% (0.44)	1.1% (0.19)	2.6% (0.22)
Lives with other adults only	15% (1.81)	31% (1.43)	43% (0.75)	22% (0.69)
Not determined	14% (2.15)	12% (0.88)	19% (0.90)	13% (0.75)

SE = standard error

^a^Population weighted

### Comparison of each health burden factor by housing type

The prevalence of health factor burden and the outcomes of the regression analyses are provided in Tables 2 and 3 respectively. Compared to participants in private rental, homeowners and homeowner mortgagees, social housing participants had significantly greater odds of reporting five of the seven health burden factors (all p values < 0.001). For example, 34% of those in social housing reported high or very high levels of distress compared to 17% of private renters, 11% of homeowners and 11% of homeowners/mortgagees. Similarly, 23% of those in social housing reported having three or more selected long-term conditions compared to 3% of private renters, 11% of homeowners and 3% of homeowners/mortgagees. Those living in social housing were more likely to report having three or more health risk behaviours compared to participants who were homeowners (p < 0.001) or homeowners/mortgagees (p = 0.016), but not compared to private rental (p = 0.363). Being overweight or obese was the only health burden factor where no significant difference was found between social housing and any other housing type (private rental p = 0.735, homeowners p = 0.219, and homeowners/mortgagees, p = 0.849).

**Table 2:** Health burden by housing type (N = 16,381,935 weighted)

	Social housing (N = 441,963, 2.7%) % (SE)^a^	Private rental (N = 3,316,040, 20.2%) % (SE)^a^	Home owner (N = 5,738,797, 35.0%) % (SE)^a^	Home owner/ mortgagee (N = 6,885,135, 42.0%) % (SE)^a^
**Self-assessed health status**				
Excellent	7% (1.27)	21% (1.10)	18% (0.67)	23% (0.73)
Very Good	15% (1.96)	36% (1.17)	34% (0.78)	39% (0.81)
Good	33% (2.42)	31% (1.00)	30% (0.76)	28% (0.65)
Fair	28% (2.31)	9% (0.74)	14% (0.59)	8% (0.42)
Poor	17% (1.74)	2.9% (0.36)	5% (0.38)	1.9% (0.22)
**Psychological distress**				
Low distress level/Unable to determine	41% (2.66)	56% (1.26)	68% (0.75)	66% (0.84)
Moderate distress level	26% (2.36)	26% (1.24)	20% (0.72)	23% (0.71)
High distress level	18% (2.21)	12% (0.83)	8% (0.55)	8% (0.44)
Very high distress level	16% (1.60)	5% (0.56)	3.4% (0.34)	3.1% (0.32)
**Disability**				
Profound/severe core activity limitation	15% (1.80)	2.6% (0.36)	6% (0.32)	2.3% (0.25)
Moderate/mild core activity limitation	44% (3.15)	15% (0.84)	28% (0.73)	13% (0.52)
No activity limitation	41% (3.12)	82% (0.92)	67% (0.75)	84% (0.57)
**Selected current long-term conditions**				
Asthma	22% (2.00)	10% (0.65)	11% (0.54)	12% (0.53)
Cancer	4.3% (1.07)	0.6% (0.16)	4.6% (0.32)	1.3% (0.19)
Kidney disease	2.8% (0.84)	0.7% (0.18)	2.2% (0.23)	0.5% (0.11)
Arthritis	36% (2.82)	9% (0.56)	33% (0.72)	13% (0.63)
Diabetes mellitus	15% (1.70)	3.9% (0.47)	10% (0.56)	3.6% (0.31)
Heart or circulatory problems	13% (1.93)	3.3% (0.40)	11% (0.46)	3% (0.26)
Mental and behavioural problems	43% (2.35)	25% (0.97)	21% (0.87)	19% (0.70)
Osteoporosis	9% (1.64)	1.6% (0.27)	10% (0.42)	2.6% (0.27)
***Mean number of selected conditions***				
0	29% (2.70)	61% (1.08)	41% (0.92)	61% (0.85)
1	31% (2.37)	27% (1.10)	31% (0.91)	27% (0.79)
2	18% (1.94)	9% (0.67)	17% (0.59)	8% (0.53)
3+	23% (2.57)	2.8% (0.39)	11% (0.48)	3.3% (0.30)
**Other long-term conditions**				
Any (yes)	57% (2.81)	27% (1.00)	49% (0.80)	32% (0.74)
Top five other conditions				
Back problems	32% (2.34)	18% (0.91)	23% (0.78)	20% (0.70)
Hypertensive disease	25% (2.18)	6% (0.53)	24% (0.73)	9% (0.40)
High cholesterol	13% (1.78)	3.5% (0.43)	14% (0.61)	5% (0.35)
COPD^b^ (bronchitis/emphysema)	11% (1.35)	2.4% (0.37)	3.9% (0.36)	2% (0.23)
Tachycardia	5% (1.13)	1.2% (0.25)	4.4% (0.31)	1.2% (0.15)
**Overweight / Obese**				
Yes	72% (3.12)	63% (1.22)	70% (0.74)	67% (0.78)
**Health risk behaviours**				
Current daily smoker	37% (2.38)	21% (0.93)	9% (0.48)	11% (0.53)
Alcohol lifetime	13% (1.96)	16% (0.84)	17% (0.71)	17% (0.62)
Alcohol short-term	30% (2.38)	50% (1.15)	31% (0.71)	50% (0.80)
Inadequate fruit and/or veg	97% (0.76)	96% (0.37)	93% (0.43)	95% (0.36)
Inadequate physical activity	91% (1.60)	85% (0.89)	83% (0.70)	85% (0.58)
***Number of health risk behaviours***				
0–1	7% (1.35)	7% (0.54)	14% (0.59)	8% (0.54)
2	47% (3.21)	44% (1.29)	55% (0.84)	47% (0.79)
3+	45% (2.95)	49% (1.21)	30% (0.77)	45% (0.81)
**Overall number health burden factors**				
0	7% (2.42)	15% (0.98)	12% (0.60)	13% (0.53)
1	15% (2.02)	32% (1.15)	26% (0.76)	32% (0.75)
2	21% (2.15)	30% (1.13)	31% (0.92)	32% (0.90)
3	17% (1.64)	14% (0.87)	17% (0.75)	15% (0.57)
4	16% (2.09)	6% (0.54)	8% (0.50)	4.7% (0.35)
5	12% (1.67)	2.5% (0.39)	4.1% (0.30)	2.2% (0.23)
6–7	12% (1.58)	1.4% (0.22)	2.1% (0.19)	0.8% (0.14)
**Mean (SE) overall health burden score**	3.1 (0.12)	1.8 (0.03)	2.0 (0.02)	1.8 (0.02)

SE = standard error

^a^Population weighted

^b^Chronic obstructive pulmonary disease

### Comparison of the overall health burden score by housing type

Those in social housing were more likely to report a higher health burden score compared to those in the other housing types (all values p < 0.001) (Table 3). For social housing participants, 24% reported the presence of five or more health burden factors compared to 3.9% for private renters, 6.2% for owners and 3% for homeowners/mortgagees. The mean burden score was 3.1 for social housing, 1.8 private renters, 2.0 for owners, and 1.8 for homeowners/mortgagees (see Table 2).

**Table 3:** Association of health burden with housing type (N = 16,381,935 weighted)

	Housing type v social housing	OR^a^	Lower CI^b^	Upper CI^b^	*p*
**Fair/poor self-assessed health**	Owner	0.26	0.21	0.32	< 0.001
	Owner mortgage	0.20	0.16	0.26	< 0.001
	Private rental	0.28	0.21	0.39	< 0.001
**High/very high distress**	Owner	0.32	0.23	0.43	< 0.001
	Owner mortgage	0.26	0.18	0.36	< 0.001
	Private rental	0.43	0.31	0.60	< 0.001
**Profound, severe or moderate disability**	Owner	0.31	0.23	0.41	< 0.001
	Owner mortgage	0.27	0.20	0.37	< 0.001
	Private rental	0.32	0.23	0.45	< 0.001
**Three or more selected conditions**	Owner	0.30	0.22	0.40	< 0.001
	Owner mortgage	0.25	0.17	0.38	< 0.001
	Private rental	0.30	0.18	0.48	< 0.001
**Any other long-term conditions**	Owner	0.52	0.41	0.66	< 0.001
	Owner mortgage	0.54	0.43	0.66	< 0.001
	Private rental	0.55	0.43	0.71	< 0.001
**Overweight or obese**	Owner	0.82	0.59	1.13	0.219
	Owner mortgage	0.97	0.70	1.34	0.849
	Private rental	0.95	0.68	1.31	0.735
**Three or more health risk behaviours**	Owner	0.58	0.45	0.76	< 0.001
	Owner mortgage	0.74	0.58	0.95	0.016
	Private rental	0.88	0.67	1.16	0.363
**Overall burden score**	Owner	0.25	0.19	0.34	< 0.001
	Owner mortgage	0.27	0.20	0.37	< 0.001
	Private rental	0.33	0.24	0.47	< 0.001

^a^OR = Odds ratio

^b^= 95% confidence interval

## Discussion

The results of this study, whilst not unexpected, point to a degree of health burden experienced by social housing tenants that is significantly higher than all other tenures. It is inevitable that those experiencing poor health and disability, and consequently dependent on pension or benefit income, will compete poorly in the private rental sector and seek accommodation in the social sector. The low incomes of social housing residents place them the wrong side of the health divide [25]. However, the ‘residualistion’ of the social housing sector resulted has amplified this health divide. In Australia, 86% of community housing and 81% of public housing allocations were to tenants defined as in ‘greatest need’ in 2020–2021 [26].

The implications for the delivery of an effective and supportive housing service are considerable, as current funding models do not reflect this context. The result is that state managed housing does little to address these issues whilst community housing providers are able to deliver more comprehensive support services based on their additional income from Commonwealth Rental Assistance (CRA) and ability to operate lower tenant to staff ratios [27]. Inevitably, without adequate funding, neither state or community housing providers can meet the growing need for tenancy support in a high needs tenant population. There are early signs of additional recognition of the challenges facing social housing providers influencing housing policy. In NSW, the Social and Affordable Housing Fund initiative has provided additional funding to community housing organisation to provide ‘coordinated tailored support services’ to with some prosects of improved social outcomes that might enable future exit to the private rental market.

The findings also point to potential policy and service reform for health service providers. The social housing population often falls into the ‘difficult to reach’ elements of the community, characterised by low health literacy, poor access to primary health services and financial inability to engage equally with diagnostic, imaging and ancillary health and wellbeing services. Late diagnosis and limited engagement with medication regimes can have significant impact on health outcomes for a wide range of conditions. In contrast, social housing services are in frequent and sustained contact with their populations, and despite the landlord/tenant relationship, generally enjoy cordial relationships with tenants. Partnerships between social housing and health services can capitalise on this relationship and target specific health interventions through outreach-based interventions [28]. Peer-led and location-based health initiatives can work particularly well where they are led by the community-based social housing team and adopt participative models of delivery [29].

There are further policy gaps in housing service delivery derived from the high incidence of mental health issues in those defined as in ‘high need’. In this study, 43% of social housing tenants indicated they had been told by a health professional that they had mental health or behavioural problem. Challenging behaviours can threaten the tenancy of the tenant and impact the safety and wellbeing of neighbouring tenancies. Challenging behaviours can result from trauma experience, addiction and diagnosed and undiagnosed mental health conditions. Better understanding of the incidence of mental health conditions can better prepare social housing providers to support tenancies and engage relevant support agencies [30]. It can also be important in recognising the causes of anti-social behaviour and designing support packages for perpetrators to avoid eviction.

Other individual health problems identified as more highly prevalent in social housing tenants compared to all other housing types included arthritis (36%), back problems (32%), hypertension (25%), asthma (22%) and COPD (11%). Along with mental health and behavioural problems, these diseases may also be a focus of support provided by social housing providers and funding allocation for health and wellbeing initiatives by social housing policy makers.

A point of interest in the findings of this study is that there was no significant difference in the prevalence of overweight and obesity between housing types. This is an unexpected given that lower socioeconomic status is often associated with higher rates of overweight and obesity [31–34]. However, in a 2008 study, Digenis-Bury et al. also found no difference in BMI between public housing and other housing types after adjusting for potential confounders [35]. These unanticipated findings might be in part explained by differences in methodologies across studies (e.g., self-reported versus objectively measured height and weight, and BMI analysed as a categorical or continuous variable) [36]. Further, a number of studies have found that in lower income populations, the positive association with overweight and obesity exists only in women or is weaker in men [32, 33]. It is also likely that the complexity of socioeconomic disadvantage is not adequately captured by the simple categorisation of social housing versus other housing tenures [37]. For example, other factors at play could include early childhood development, social inclusion, or discrimination- data not collected by the ABS questionnaire.

The limitations and strengths of the study should be considered when interpreting the findings. The definition used to identify social housing tenants in theory excludes community housing tenants. However, given the contractual relationship of community housing organisations with state government departments, tenants are often unable to make the distinction. The health factors examined by the research were limited by the survey items included in the ABS questionnaire, and so some important health factors may have been excluded. A strength of the study is the large representative sample.

## Conclusions

In summary, this study confirmed the high prevalence of health burden experience by social housing tenants and demonstrated the high overall health burden experienced by this group compared to other housing type. Social housing providers as well as policy and decision makers in housing and health should consider the study findings when planning future initiatives. This could include changes in social housing funding models to so providers can better support social housing tenant’s multimorbidity. Partnerships could be built between social housing and health services to target the prevention and management of chronic disease. Further, partnerships with other sectors, such education and employment, could help to address the social determinants of poor health and wellbeing experience by social hosing tenants.

## Data Availability

*Use of an existing database*: Some summary statistics from the National Health Survey (NHS) 2017–18 dataset are publicly available from the Australian Bureau of Statistics (ABS). However, those seeking access to the data used for the type of research outlined in the current study would need to seek approval for virtual access to the detailed microdata files that remain in the secure ABS environment. Prior to the survey interview, respondents were provided a guide that described their selection for the survey and information regarding the background to the survey, interview process and confidentiality provisions under the Census and Statistics Act 1905. Selected Individuals were requested requested to answer the questions, but if the Australian Statistician directs an individual to provide the information, they were legally obliged to do so. All ABS surveys comply with the requirements of the Privacy Act 1988. The datasets used and/or analysed during the current study are available from the corresponding author on reasonable request.

## References

[1] Organisation for Economic Co-operation and Development. Social housing: A key part of past and future housing policy [Internet]. Paris: OECD. ; 2020. Available from: http://oe.cd/social-housing-2020.

[2] Clarke A, Cheshire L, Parsell C, Morris A (2022). Reified scarcity & the problem space of ‘need’: unpacking Australian social housing policy. Hous Stud.

[3] Chen J, Stephens M, Man Y, editors. The Future of Public Housing [Internet]. Berlin, Heidelberg: Springer Berlin Heidelberg; 2013 [cited 2022 May 27]. Available from: http://link.springer.com/10.1007/978-3-642-41622-4.

[4] Bentley R, Baker E, Simons K, Simpson JA, Blakely T (2018). The impact of social housing on mental health: longitudinal analyses using marginal structural models and machine learning-generated weights. Int J Epidemiol.

[5] Taylor AW, Pilkington R, Grande ED, Kourbelis C, Barry H (2019). Health and welfare profile of Australian baby boomers who live in rented accommodation – implications for the future. Ageing Soc.

[6] Holding E, Blank L, Crowder M, Ferrari E, Goyder E (2020). Exploring the relationship between housing concerns, mental health and wellbeing: a qualitative study of social housing tenants. J Public Health.

[7] Sharpley CF, Murcell N, Anderson M, Bitsika V, Fourie PJ, Agnew LL (2021). An exploration of recent life stress, psychological resilience, purpose in life, and optimism as correlates of depression in social housing residents in rural Australia. Int J Mental Health.

[8] Koné Pefoyo AJ, Bronskill SE, Gruneir A, Calzavara A, Thavorn K, Petrosyan Y (2015). The increasing burden and complexity of multimorbidity. BMC Public Health.

[9] Fortin M, Bravo G, Hudon C, Lapointe L, Almirall J, Dubois MF (2006). Relationship between multimorbidity and health-related quality of life of patients in primary care. Qual Life Res.

[10] Barnett K, Mercer SW, Norbury M, Watt G, Wyke S, Guthrie B (2012). Epidemiology of multimorbidity and implications for health care, research, and medical education: a cross-sectional study. The Lancet.

[11] Fortin M, Haggerty J, Almirall J, Bouhali T, Sasseville M, Lemieux M (2014). Lifestyle factors and multimorbidity: a cross sectional study. BMC Public Health.

[12] Salive ME (2013). Multimorbidity in older adults. Epidemiol Rev.

[13] Tucker-Seeley RD, Li Y, Sorensen G, Subramanian S (2011). Lifecourse socioeconomic circumstances and multimorbidity among older adults. BMC Public Health.

[14] Adamson D. Two modes of co-production in social housing: comparing UK and Australian experience. Affordable housing governance and finance. Routledge; 2018. pp. 144–64.

[15] Australian Bureau of Statistics. National Health Survey: First results, 2017-18 financial year [Internet]. 2018 [cited 2020 Aug 1]. Available from: https://www.abs.gov.au/statistics/health/health-conditions-and-risks/national-health-survey-first-results/latest-release.

[16] Australian Bureau of Statistics. 4363.0 - National Health Survey: Users’ Guide, 2017-18 [Internet]. c = AU; o = Commonwealth of Australia; ou = Australian Bureau of Statistics; 2019 [cited 2021 Aug 1]. Available from: https://www.abs.gov.au/AUSSTATS/abs@.nsf/DetailsPage/4363.02017-18?OpenDocument.

[17] Australian Bureau of Statistics. 4363.0 - National Health Survey: Users’ Guide, 2017-18: Kessler Psychological Distress Scale – 10 (K10) [Internet]. c = AU; o = Commonwealth of Australia; ou = Australian Bureau of Statistics; 2019 [cited 2021 Aug 1]. Available from: https://www.abs.gov.au/ausstats/abs@.nsf/Lookup/by%20Subject/4363.0~2017-18~Main%20Features~Kessler%20Psychological%20Distress%20Scale%20-10%20(K10)~35.

[18] Commonwealth of Australia. The National Obesity Strategy 2022–2032 [Internet]. Health Ministers Meeting. ; 2022. Available from: https://www.health.gov.au/sites/default/files/documents/2022/03/national-obesity-strategy-2022-2032_0.pdf.

[19] Australian Bureau of Statistics. 4363.0 - National Health Survey: Users’ Guide, 2017-18: Alcohol consumption [Internet]. c = AU; o = Commonwealth of Australia; ou = Australian Bureau of Statistics; 2019 [cited 2021 Aug 1]. Available from: https://www.abs.gov.au/ausstats/abs@.nsf/Lookup/by%20Subject/4363.0~2017-18~Main%20Features~Alcohol%20consumption~40.

[20] Australian Bureau of Statistics. 4363.0 - National Health Survey: Users’ Guide, 2017-18: Fruit and vegetable consumption [Internet]. c = AU; o = Commonwealth of Australia; ou = Australian Bureau of Statistics; 2019 [cited 2021 Aug 1]. Available from: https://www.abs.gov.au/ausstats/abs@.nsf/Lookup/by%20Subject/4363.0~2017-18~Main%20Features~Fruit%20and%20vegetable%20consumption~42.

[21] Australian Bureau of Statistics. 4363.0 - National Health Survey: Users’ Guide, 2017-18: Physical activity [Internet]. c = AU; o = Commonwealth of Australia; ou = Australian Bureau of Statistics; 2019 [cited 2021 Aug 1]. Available from: https://www.abs.gov.au/ausstats/abs@.nsf/Lookup/by%20Subject/4363.0~2017-18~Main%20Features~Physical%20activity~41.

[22] Australian Bureau of Statistics. 2033.0.55.001 - Census of Population and Housing: Socio-Economic Indexes for Areas (SEIFA), Australia, 2016 [Internet]. c = AU; o = Commonwealth of Australia; ou = Australian Bureau of Statistics; 2018 [cited 2021 Aug 1]. Available from: https://www.abs.gov.au/AUSSTATS/abs@.nsf/Lookup/2033.055.001Main+Features12016?OpenDocument.

[23] Australian Bureau of Statistics. 1270.0.55.005 - Australian Statistical Geography Standard (ASGS): Volume 5 - Remoteness Structure, July 2016 [Internet]. c = AU; o = Commonwealth of Australia; ou = Australian Bureau of Statistics; 2018 [cited 2021 Aug 1]. Available from: https://www.abs.gov.au/AUSSTATS/abs@.nsf/Latestproducts/1270.055.005Main%20Features20July%202016.

[24] SAS Institute Inc. SAS 9.4 Software [Internet]. Cary, NC, USA. ; 2013 [cited 2022 Jun 16]. Available from: https://support.sas.com/software/94/.

[25] Rolfe S, Garnham L, Godwin J, Anderson I, Seaman P, Donaldson C (2020). Housing as a social determinant of health and wellbeing: developing an empirically-informed realist theoretical framework. BMC Public Health.

[26] Australian Government Productivity Commission. Report on Government Services 2022: 18 Housing [Internet]. 2022 [cited 2021 Aug 1]. Available from: https://www.pc.gov.au/research/ongoing/report-on-government-services/2022/housing-and-homelessness/housing.

[27] Flanagan K, Levin I, Tually S, Varadharajan M, Verdouw J, Faulkner D et al. Understanding the experience of social housing pathways. AHURI Final Report [Internet]. 2019 Dec [cited 2022 Jun 8];(324). Available from: http://www.ahuri.edu.au/research/final-reports/324.

[28] Hernández D (2019). Housing-based Health interventions: harnessing the Social Utility of Housing to Promote Health. Am J Public Health.

[29] Srivarathan A, Lund R, Christensen U, Kristiansen M (2020). Social relations, Community Engagement and potentials: a qualitative study exploring Resident Engagement in a community-based Health Promotion intervention in a deprived Social Housing Area. Int J Environ Res Public Health.

[30] Atkinson RG, Habibis D, Easthope H, Goss DN. Sustaining tenants with demanding behaviour: a review of the research evidence. In: Positioning Paper; Australian Housing and Urban Research Institute [Internet]. University of NSW, Sydney: AHURI; 2007 [cited 2022 Jun 8]. p. Jan-47. Available from: http://www.ahuri.edu.au/publications/projects/p40327.

[31] Stunkard A, Cardew G. Socioeconomic status and obesity. Origins and consequences of obesity. John Wiley & Sons; 1996. pp. 174–87.

[32] Merino Ventosa M, Urbanos-Garrido RM (2016). Disentangling effects of socioeconomic status on obesity: a cross-sectional study of the Spanish adult population. Econ Hum Biology.

[33] Newton S, Braithwaite D, Akinyemiju TF (2017). Socio-economic status over the life course and obesity: systematic review and meta-analysis. PLoS ONE.

[34] Australian Institute of Health and Welfare. Inequalities in overweight and obesity and the social determinants of health 2007–08 to 2017–18 [Internet]. AIHW. ; 2021 [cited 2022 Aug 1]. Available from: https://www.aihw.gov.au/getmedia/9cc2f996-cf45-4769-bfc7-20026a893ef9/aihw-phe-278.pdf.aspx?inline=true.

[35] Digenis-Bury EC, Brooks DR, Chen L, Ostrem M, Horsburgh CR (2008). Use of a Population-based Survey to describe the Health of Boston Public Housing Residents. Am J Public Health.

[36] Ng CD (2019). Biases in self-reported height and weight measurements and their effects on modeling health outcomes. SSM Popul Health.

[37] Ball K, Mishra G, Crawford D (2002). Which aspects of socioeconomic status are related to obesity among men and women?. Int J Obes.

